# Supplementary material for: Democratising “Microscopi”: a 3D printed automated XYZT fluorescence imaging system for teaching, outreach and fieldwork
Source: Wellcome Open Res. 2022 Mar 30;6:63. Originally published 2021 Mar 22. [Version 2] doi: 10.12688/wellcomeopenres.16536.2 (PMC8082569; doi:10.12688/wellcomeopenres.16536.2)
Supplement: Peer review history

# Peer review: Reviewer response for version 1

**DOI:** 10.21956/wellcomeopenres.18222.r43201

**Published:** 2021-04-28

**Authors:** Kerry Thompson

**Affiliations:** 1 Centre for Microscopy and Imaging, Anatomy, School of Medicine, National University of Ireland, Galway, Ireland

This manuscript describes in explicit detail the design, construction and use of an open source microscope who's applications include research, interdisciplinary teaching and outreach.The authors highlight the cost effect design strategy and even go as far as to having considered the environmental impacts and recycling of the build components' printed frame. Furthermore, "soldering free" options, for the often more complicated electronic component assembly, are presented in as far as is practical. This truly acknowledges the multidisciplinary user base the system is intended to reach. The comprehensive workflow should encourage educators interested in developing and deploying more open source systems in their teaching and research to attempt this clearly explained design.  

 Comments and Suggestions:

- I agree with reviewer one - would it be possible to include some more information or specifics of the objectives used in the main body of text (Part numbers for  lenses are also not mentioned in the Assembly Guide).
- Could the authors suggest some other simple experiments, activities or suitable samples for those wishing to carry out outreach with the system?
- As a final suggestion - Could the authors perhaps reword the following "Fly parts were obtained from individual flies from lab-cultured *Drosophila*, killed by extended exposure to CO _2_" to ""Fly parts were obtained from individual flies from lab-cultured *Drosophila*, euthanized by extended exposure to CO _2_".

Is the rationale for developing the new method (or application) clearly explained?

Yes

Is the description of the method technically sound?

Yes

Are the conclusions about the method and its performance adequately supported by the findings presented in the article?

Yes

If any results are presented, are all the source data underlying the results available to ensure full reproducibility?

Yes

Are sufficient details provided to allow replication of the method development and its use by others?

Yes

Reviewer Expertise:

Biological microscopy & imaging, histology & cell biology, education & outreach, open source technologies.

I confirm that I have read this submission and believe that I have an appropriate level of expertise to confirm that it is of an acceptable scientific standard.

# Peer review: Reviewer response for version 1

**DOI:** 10.21956/wellcomeopenres.18222.r43384

**Published:** 2021-04-19

**Authors:** Simon Bullock

**Affiliations:** 1 Division of Cell Biology, MRC Laboratory of Molecular Biology, Cambridge, UK

This manuscript describes the development and design of an economical automated bright field and fluorescence imaging system, and provides detailed guidance into its construction and use. Although other budget microscopes have been developed, principally for educational or field work, they tend to compromise on image quality or automation. Systems that do not suffer from these limitations tend to cost an order of magnitude more than the 'Microscopi' system described here. The Microscopi system therefore fills a valuable niche and is likely to have a substantial impact. The use of Raspberry Pi and Python will increase the value of the system as a teaching tool.

 The manuscript is very well written and the utility of the system is nicely supported by data from different imaging modalities. I have no substantial criticisms and only offer the following minor suggestions for improving the clarity of the work:

1. Users are likely to be sufficiently interested in the details of the objectives (numerical aperture, colour correction etc) for this to feature in the main document (not just the supplementary material).
2. It would be helpful to include which objectives were used to capture which images in the legends. This will help readers judge the quality of images produced with each objective.
3. In the introduction, it was unclear why the system described by Diederich *et al.* "may be limited in its application to the wider range of imaging subjects". The authors should clarify why this is likely to be case.
4. In the Summary, the authors describe future adaptations of the system to address the applications they have in mind. It would be helpful to expand briefly on what these adaptations are as this could highlight potential limitations with the current set up.
5. Optional: It would be helpful to have further information on the sensitivity of the instrument for fluorescence imaging if the authors already have data available. For example, can Microscopi image tissue culture cells stained with phalloidin or other vital stains, which is something many biological users will be familiar with as a reference?

Is the rationale for developing the new method (or application) clearly explained?

Yes

Is the description of the method technically sound?

Yes

Are the conclusions about the method and its performance adequately supported by the findings presented in the article?

Yes

If any results are presented, are all the source data underlying the results available to ensure full reproducibility?

Yes

Are sufficient details provided to allow replication of the method development and its use by others?

Yes

Reviewer Expertise:

cell biology; imaging; microtubules; motors; Drosophila; cytoskeleton; neuronal cell biology; schools outreach

I confirm that I have read this submission and believe that I have an appropriate level of expertise to confirm that it is of an acceptable scientific standard.
